# A Rare Presentation of Small Bowel Volvulus Secondary to Meckel’s Diverticulum-Induced Intussusception in an Adolescent Female

**DOI:** 10.7759/cureus.100364

**Published:** 2025-12-29

**Authors:** David Heath, Natasha Patrick, Christine Li, Fiona Lee

**Affiliations:** 1 Department of General Surgery, Bunbury Regional Hospital, Bunbury, AUS; 2 Department of General Surgery, Launceston General Hospital, Launceston, AUS

**Keywords:** appendicitis, cecal intussusception, meckel's diverticulum, small bowel volvulus, volvulus

## Abstract

Meckel’s diverticulum is the most common congenital anomaly of the gastrointestinal tract, yet it remains asymptomatic in the majority of cases. When complications occur, they typically include bleeding, obstruction, or inflammation. The combination of intussusception leading to a subsequent small bowel volvulus is an exceedingly rare presentation, particularly in the adolescent population, and poses a significant diagnostic challenge, often mimicking more common conditions such as acute appendicitis. We present the case of a 15-year-old female who presented to the emergency department with a one-day history of sudden-onset, severe right iliac fossa (RIF) pain. She had no associated nausea, vomiting, or changes in bowel habits. Physical examination revealed localized tenderness in the RIF. An initial abdominal ultrasound reported a cecal intussusception with reactive mesenteric lymph nodes, and the appendix was not visualized. Given the clinical and radiological findings, the patient was taken to the operating theater for a diagnostic laparoscopy. Intraoperatively, an ileoileal intussusception was identified, which, upon reduction, revealed a Meckel’s diverticulum acting as the lead point. This segment had also caused a 360-degree midgut volvulus. The volvulus was detorsed, and the bowel was found to be viable. The procedure was completed with resection of the Meckel’s diverticulum and transverse closure of the antimesenteric border of the small bowel via a mini-laparotomy, along with an incidental appendectomy. The patient had an uneventful postoperative recovery and was discharged on day 7. This case highlights a rare and complex cause of acute abdominal pain in an adolescent. It underscores the importance of maintaining a high index of suspicion for complicated Meckel’s diverticulum in young patients presenting with features of intussusception or atypical appendicitis. Early surgical intervention is crucial to prevent bowel ischemia and associated morbidity. Laparoscopy proved to be an invaluable tool for both diagnosis and initial management in this case.

## Introduction

Meckel’s diverticulum is a remnant of the omphalomesenteric duct and represents the most prevalent congenital anomaly of the gastrointestinal tract [1]. It is often cited to follow the “Rule of 2s”: occurring in approximately 2% of the population, located within two feet (about 60 cm) of the ileocecal valve, typically measuring two inches (about 5 cm) in length, and most commonly presenting before the age of two [2,3]. While the majority of individuals with a Meckel’s diverticulum remain asymptomatic throughout their lives, a lifetime complication rate of 4-6% has been reported [4].

Complications arising from a Meckel’s diverticulum are diverse and include gastrointestinal bleeding (most common in children, often due to ectopic gastric mucosa), small bowel obstruction, diverticulitis, and perforation [5,6]. Small bowel obstruction can occur through several mechanisms, including volvulus around a vitelline band, herniation (Littre’s hernia), or intussusception, in which the diverticulum acts as a pathological lead point [7].

Intussusception in adolescents and adults is rare, accounting for only 5% of all cases. Unlike idiopathic pediatric cases, it is typically secondary to a pathological lead point: benign in adolescents but often malignant in adults, with up to 90% of adult cases having an identifiable pathological cause [8,9]. Meckel’s diverticulum as the lead point is a well-described, albeit uncommon, cause. The subsequent development of a small bowel volvulus secondary to this intussusception is an even rarer event. This dual pathology, intussusception and volvulus, presents a complex surgical emergency that, if untreated, can rapidly progress to ischemia, necrosis, and perforation, often necessitating bowel resection [10].

The clinical presentation is often nonspecific, with acute abdominal pain being the primary symptom, frequently mimicking more common conditions such as acute appendicitis. This diagnostic ambiguity can lead to delays in definitive management [11]. Imaging, particularly CT, is often utilized in the work-up of undifferentiated abdominal pain, but ultrasound remains a valuable first-line investigation in younger patients, as in our case [12].

This paper presents the case of a 15-year-old female with an acute abdomen, initially suspected to be appendicitis or cecal intussusception on ultrasound, who was found intraoperatively to have a small bowel volvulus secondary to an ileoileal intussusception caused by a Meckel’s diverticulum. We aim to discuss the diagnostic challenges and surgical management of this rare presentation, supplemented by a review of relevant contemporary literature.

## Case presentation

A 15-year-old female with no significant past medical or surgical history presented to the emergency department with a 24-hour history of sudden-onset, sharp, nonradiating pain localized to the RIF. The pain was constant and rated 8/10 in severity. She denied nausea, vomiting, fever, or changes in bowel or urinary habits. Her last menstrual period was two weeks prior.

On physical examination, she was afebrile and hemodynamically stable. Abdominal examination revealed localized tenderness and guarding in the RIF, with a positive Rovsing’s sign and tenderness at McBurney’s point. There were no palpable masses or signs of generalized peritonitis.

Initial laboratory investigations showed a normal white blood cell count of 8.5 × 10⁹/L (reference range: 4.0-11.0 × 10⁹/L) and a CRP of <5 mg/L (reference range: <5 mg/L). A urine pregnancy test was negative (Table 1).

**Table 1:** Initial laboratory investigation results

Laboratory parameter	Result	Reference range	Interpretation
WBC count	8.5 × 10⁹/L	4.0-11.0 × 10⁹/L	Normal
CRP	<5 mg/L	<5 mg/L	Normal
Urine pregnancy test	Negative	Negative (nonpregnant)	Normal

Given the strong clinical suspicion of acute appendicitis, an urgent abdominal and pelvic ultrasound was performed. The sonographer reported features suggestive of a cecal intussusception, visualized as a “target sign” in the RIF. The appendix was not identified, and a moderate number of reactive mesenteric lymph nodes were noted. There was no evidence of free fluid or other acute pathology.

Based on the clinical findings and the ultrasound report of a potential intussusception, a decision was made for urgent surgical exploration via diagnostic laparoscopy to establish a definitive diagnosis and guide management.

The patient was taken to the operating theater and underwent diagnostic laparoscopy under general anesthesia. A 10 mm umbilical port was inserted using the Hasson technique, followed by a 5 mm port in the left iliac fossa and a 5 mm suprapubic port. Initial inspection of the pelvis and RIF revealed a normal-appearing appendix and cecum. The appendix was removed laparoscopically, as per standard practice at our institution in cases of negative appendicitis exploration.

**Figure 1:**
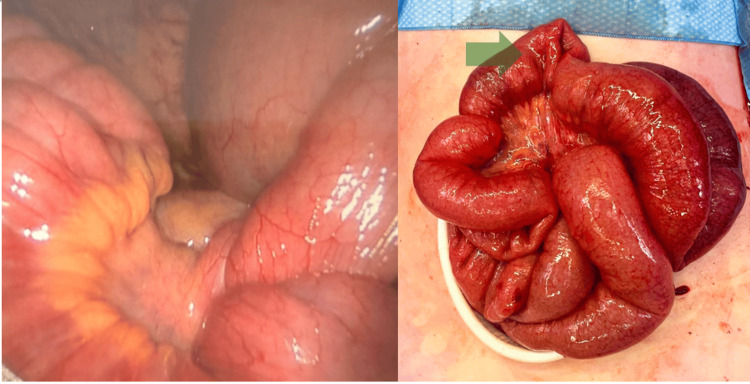
Intraoperative laparoscopic view demonstrating the volvulus The image shows the segment of the proximal ileum (intussusceptum) telescoped into the distal ileum (intussuscipiens). The Meckel’s diverticulum, acting as the lead point, is located at the apex of the intussusceptum, though it is not directly visible in this pre-reduction view. The bowel appears congested but viable.

Upon running the small bowel proximally from the ileocecal valve, a segment of dilated, congested proximal small bowel was encountered. Further exploration revealed a 360-degree clockwise volvulus of the mid-ileum. Attempts to laparoscopically detorse the volvulus were unsuccessful, and the umbilical port site was extended into a 10 cm mini-laparotomy longitudinal incision. The volvulus was carefully detorsed, and an underlying ileoileal intussusception was identified approximately 60 cm from the ileocecal valve (Figure 1). The lead point was identified as a broad-based, inflamed Meckel’s diverticulum. After successful reduction, the bowel was viable.

The Meckel’s diverticulum underwent wedge resection at its base on the antimesenteric border, and the small bowel was closed in a transverse fashion with a hand-sewn anastomosis to avoid narrowing the lumen. The bowel was returned to the abdominal cavity, and the incisions were closed in layers.

The patient had an uneventful postoperative recovery. She was started on clear fluids on postoperative day 1, and her diet was gradually advanced. She experienced an expected postoperative ileus, with bowel movements resuming by day 5. Pain was managed with an oxycodone patient-controlled analgesia, and she was gradually transitioned to oral analgesics. She was discharged home on postoperative day 7.

Histopathology confirmed a Meckel’s diverticulum with mucosal ulceration and the presence of ectopic gastric and pancreatic tissue. The appendix was reported as normal. At a six-week follow-up appointment, she was asymptomatic and had made a full recovery.

## Discussion

This case illustrates a highly unusual and complex sequence of surgical pathologies: a Meckel’s diverticulum causing an ileoileal intussusception, which in turn precipitated a small bowel volvulus. The presentation, mimicking acute appendicitis, highlights the significant diagnostic challenges that can arise with complications of Meckel’s diverticulum.

Meckel’s diverticulum arises from the incomplete obliteration of the vitelline duct, a structure connecting the yolk sac to the midgut during embryonic development [1]. While most remain asymptomatic, the lifetime risk of developing a complication is estimated at approximately 4-6% [4]. In children, the most common complication is bleeding from ectopic gastric mucosa within the diverticulum, whereas in adults, bowel obstruction is the most frequent presentation [13].

Intussusception caused by a Meckel’s diverticulum occurs when the diverticulum inverts into the lumen of the small bowel and is propelled distally by peristalsis, acting as a lead point that telescopes the proximal bowel into the distal segment [14]. This is more common in children than in adults. The development of a secondary volvulus is a rare and dangerous progression. It is postulated that the mass effect of the intussusceptum, combined with its mobile mesentery, can create a pivot point around which the bowel twists, leading to closed-loop obstruction and compromised vascular supply [10,15]. The risk of bowel ischemia increases significantly when these two obstructive pathologies coexist.

The primary challenge in this case was the preoperative diagnosis. The patient’s symptoms, such as acute RIF pain, localized tenderness, and a positive Rovsing's sign, created a strong clinical suspicion of acute appendicitis, the most common surgical emergency in this age group [11]. The absence of nausea, vomiting, or significant inflammatory marker elevation made the presentation slightly atypical but not sufficient to dismiss appendicitis as the primary differential.

Ultrasound is a key initial imaging modality for suspected appendicitis in young patients, primarily to avoid the ionizing radiation of a CT scan. While it has high sensitivity and specificity for appendicitis, its utility in diagnosing other pathologies can be operator-dependent [16]. In this instance, the ultrasound correctly identified an intussusception but localized it to the cecum (ileocolic), the most common type in young children but less frequent in adolescents. Intraoperatively, the intussusception was ileoileal. This discrepancy is understandable, as inflamed bowel loops in the RIF can be difficult to localize precisely. Importantly, the ultrasound identified a significant pathology requiring surgical intervention, guiding clinical decision-making appropriately, even if the exact anatomical details were clarified only intraoperatively.

CT scans offer superior anatomical detail and are often the investigation of choice for suspected bowel obstruction or diagnostic uncertainty in adults, but their use is more selective in the pediatric and adolescent populations [12,17]. The decision to proceed with diagnostic laparoscopy was critical. Laparoscopy is widely accepted for both diagnosing and managing suspected appendicitis and allows full exploration of the abdominal cavity when the appendix appears normal [18]. In this case, it was instrumental in correctly identifying the dual pathology of volvulus and intussusception and assessing bowel viability in a minimally invasive manner.

Once the diagnosis was established, several management decisions were required. Reduction vs. resection is a primary consideration in intussusception with a pathological lead point. Some surgeons advocate en bloc resection without reduction, particularly in adults, due to the risk of underlying malignancy and theoretical concerns of tumor seeding or perforation [8]. However, in a young patient where malignancy is unlikely and the lead point is benign, gentle reduction is often preferred to preserve bowel length, especially if the bowel appears viable [19]. In this case, reduction was achievable via the mini-laparotomy incision.

Wedge resection vs. segmental resection is another consideration. The choice depends on the characteristics of the diverticulum and adjacent bowel. Wedge resection is appropriate for diverticula with a narrow base, no inflammation at the base, and no signs of ischemia in the adjacent ileum [20]. Segmental resection with primary anastomosis is indicated for diverticula with a broad base, evidence of inflammation or ischemia at the junction with the ileum, or if ectopic tissue at the base could be transected by a stapler [3,7]. In this case, despite a broad base, the tissue was healthy, allowing for a safe wedge resection via mini-laparotomy with secure closure.

Incidental appendectomy, the removal of a normal appendix during surgery for another condition, is a common practice, particularly in younger patients, to prevent future diagnostic confusion should the patient develop RIF pain again. This remains a standard approach in many centers across Australia and New Zealand.

## Conclusions

Small bowel volvulus secondary to intussusception from a Meckel’s diverticulum is a rare but potentially life-threatening cause of acute abdomen in adolescents. This case contributes to the limited literature on this specific combination of pathologies and underscores the need for a high index of suspicion for Meckel’s diverticulum complications in young patients presenting with acute abdominal pain, especially when clinical or radiological findings are atypical for common conditions such as appendicitis. The successful outcome in our patient highlights the pivotal role of prompt diagnostic laparoscopy in accurately identifying complex intra-abdominal pathology and guiding appropriate, stepwise, minimally invasive surgical management to prevent catastrophic bowel compromise.
